# ﻿On the identity of Thymushumifususvar.aureopunctatus (Lamiaceae) and taxonomic notes on the *Th.richardii* complex

**DOI:** 10.3897/phytokeys.186.75412

**Published:** 2021-12-09

**Authors:** Llorenç Sáez, Faruk Bogunić, Salvatore Cambria, Jesús Riera, Sandro Bogdanović

**Affiliations:** 1 Systematics and Evolution of Vascular Plants (UAB) – Associated Unit to CSIC, Departament de Biologia Animal, Biologia Vegetal i Ecologia, Facultat de Biociències, Universitat Autònoma de Barcelona, 08193 Bellaterra, Spain Universitat Autònoma de Barcelona Barcelona Spain; 2 University of Sarajevo, Faculty of Forestry, Zagrebačka 20, 71 000 Sarajevo, Bosnia and Herzegovina University of Sarajevo Sarajevo Bosnia and Herzegovina; 3 Department of Biological, Geological and Environmental Sciences, University of Catania, Via A. Longo 19, 95125 Catania, Italy University of Catania Catania Italy; 4 Jardín Botánico. Universidad de Valencia. C/ Quart, 80. 46008 Valencia, Spain Universidad de Valencia Valencia Spain; 5 University of Zagreb, Faculty of Agriculture, Department of Agricultural Botany, Svetošimunska 25, 10000 Zagreb, Croatia University of Zagreb Zagreb Croatia; 6 Centre of Excellence for Biodiversity and Molecular Plant Breeding, Svetošimunska 25, 10000 Zagreb, Croatia Centre of Excellence for Biodiversity and Molecular Plant Breeding Zagreb Croatia

**Keywords:** Balkan Peninsula, Mediterranean, nomenclature, original material, taxonomy, typification

## Abstract

The name Thymushumifususvar.aureopunctatus, described from Bosnia and Herzegovina, is lectotypified, and its taxonomic value is discussed. Thymusrichardiisubsp.richardii is currently considered an endemic subspecies common to Mallorca (Balearic Islands) and Bosnia and Herzegovina from the Balkan Peninsula. Specimens identified as *Th.richardii* from both Balearic Islands and Bosnia and Herzegovina were studied to determine if they are indeed the same taxonomic entity. Detailed micromorphological observations and morphometric analysis, suggest that the Balkan plants (Th.humifususvar.aureopunctatus) and the Majorcan populations (Th.richardiisubsp.richardii) are clearly separate entities. For the former name, based on morphological, phytochemical, biogeographical and present results, we propose the subspecific rank, as Th.richardiisubsp.aureopunctatus**comb. & stat. nov.** Full descriptions of all five subspecies currently accepted within *Th.richardii* are provided.

## ﻿Introduction

The western Mediterranean Basin is one of the most important regions where the genus *Thymus* L. has diversified (Morales 1997). Thymussect.Serpyllum (Mill.) Benth. is a difficult group taxonomically that includes the largest number of species of the genus (Morales 1997). One of the species included in this section is *Thymusrichardii* Pers. a diploid (2*n* = 28, 30) Mediterranean species with a strongly fragmented distribution (Morales 1997, 2010; Bartolucci 2018; Bartolucci et al. 2018). This species represents an aggregate of allopatric subspecies: Th.richardiisubsp.richardii (2*n* = 28, 30), occurring in Mallorca and Bosnia and Herzegovina (Balkans), Th.richardiisubsp.ebusitanus (Font Quer) Jalas, endemic to Ibiza (2*n* = 30), Th.richardiisubsp.vigoi Riera, Güemes & Rosselló, endemic to eastern Spain (Valencia and Alicante provinces) and Th.richardiisubsp.nitidus (Guss.) Jalas, endemic of Marettimo Island (Sicily; 2*n* = 28) (Riera et al. 2007; Morales 2010; Bartolucci et al. 2013; Brullo and Brullo 2020).

As currently circumscribed, Th.richardiisubsp.richardii presents a striking distribution pattern, since the isolation between both areas (Mallorca and the Bosnia) is remarkable, and there are no other cases of shared endemism between these areas. Furthermore, i) the differences between the habitat occupied by Th.richardiisubsp.richardii in both areas, ii) the differences in the composition of essential oils (Llorens et al. 2014), and iii) the fact that the Bosnian and Herzegovinian population was initially recognised as a separate taxon (Th.humifususvar.aureopunctatus Beck) by several authors (Malý 1908, 1923; Ronniger 1930a, b) invites a re-evaluation of the inclusion of the latter taxon within the synonymy of Th.richardiisubsp.richardii as originally proposed by Jalas (1971).

In order to elucidate the taxonomic identity of Th.humifususvar.aureopunctatus, we have sampled specimens from the Bosnian and Herzegovinian and Balearic populations of *Th.richardii* in the field for a detailed comparison. On the other hand, the morphological characters used to separate the rest of the subspecies recognised in *Th.richardii* have also been analysed in detail. Finally, a multivariate morphometric analysis based on quantitative traits was carried out to clarify the relationships among taxa within the *Th.richardii* complex.

## ﻿Material and methods

### ﻿Plant material

This study is based on analysis of relevant literature, field surveys and examination of herbarium specimens kept in BC, BCN, COI, HBJS, MA, P, PAL, SARA, VAL, ZA, ZAGR (herbarium codes according to Thiers 2021) and the Herbarium of the Balearic Islands University. For the typification purposes, herbarium specimens deposited at BC, BM, L, P, and PRC were studied using the online herbarium databases or images were requested.

**Figure 1.**
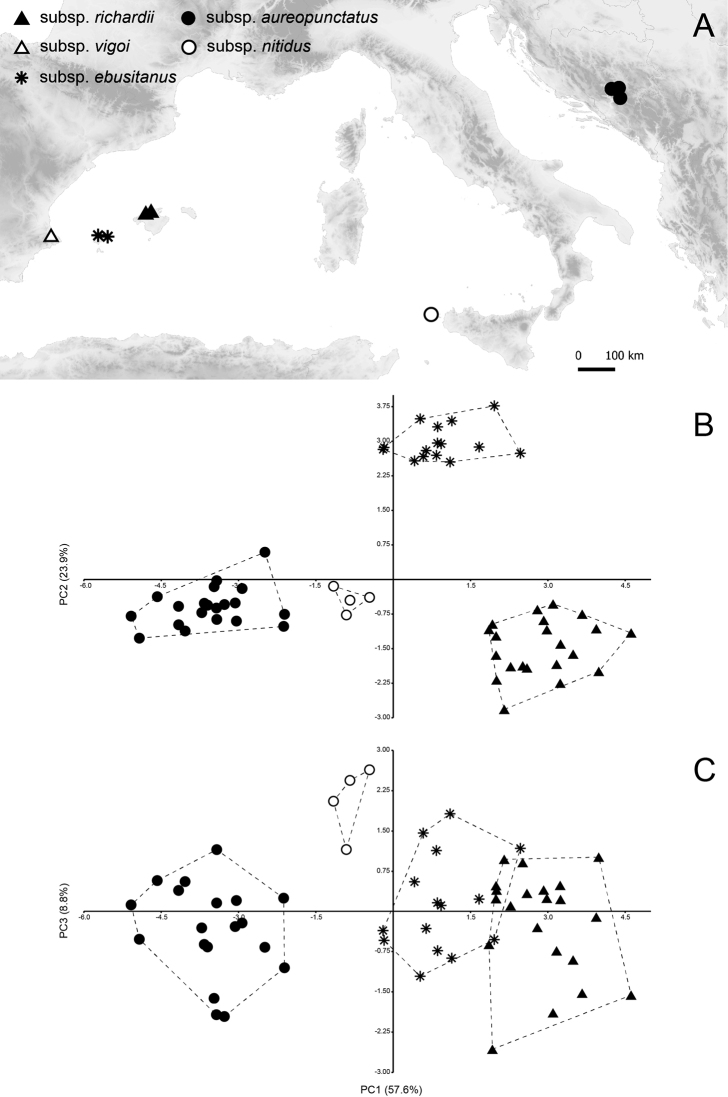
Principal Component Analysis (PCA) of 61 individuals of the *Thymusrichardii* complex **A** distribution of the studied samples **B** plot of analysed samples by first and second principal components. **C** plot of analysed samples by first and third principal components.

Furthermore, the plant material of recently collected samples of Th.humifususvar.aureopunctatus and Th.richardiisubsp.richardii from Bosnia and Herzegovina and Mallorca was analysed, too (Fig. 1A). In total, 61 individuals from seven populations (see Appendix 1) were surveyed for micromorphology and quantitative morphometry.

Morphological characters recognised as taxonomically discriminant within the *Th.richardii* complex (Jalas 1972; Morales 2010; and our own observations) were scored either in the field or in the herbarium specimens (Table 1, 3). Morphological observations of materials were carried out under a Zeiss Stemi DV4 binocular stereoscopic microscope. We scored both qualitative and quantitative traits in evaluation of taxa, the latter ones were used to describe the pattern of morphological variation and relationships among taxa.

**Table 1.** Descriptive parameters of the analysed traits of the *Thymusrichardii* complex: minimum and maximum values in brackets, mean value with standard deviations and coefficients of variation (%) in brackets.

Trait	subsp. richardii	subsp. aureopunctatus	subsp. ebusitanus	subsp. nitidus	subsp. vigoi
leaf base	cuneate	cuneate	cuneate	cuneate	cordate
leaf margin	entire	entire	entire	entire	denticulate
leaf (blade) length (mm)	(7.0–2.03) 9.45 ± 1.40 (14.81)	(4.83–7.4) 6.22 ± 0.72 (13.74)	(7.0–12.03) 8.62 ± 1.40 (14.80)	(6.43–9) 7.66 ±1.05 (13.74)	(7.4–9.738.62 ± 1.17 (13.57)
leaf width (mm)	(4.3–7.07) 5.52 ± 0.93 (16.85)	(3.06–4.93) 3.67 ± 0.59 (15.40)	(4.53–6.23) 5.64 ± 0.46 (8.18)	(3.1–4.1) 3.67 ±0.43 (11.83)	(5.76–7.96) 6.53 ± 1.24 (19.01)
ratio LL/LW	(1.21–2.14) 1.73 ± 0.22 (12.96)	(1.34–2.01) 1.62 ± 0.17 (10.55)	(1.23–1.87) 1.53 ± 0.21 (13.84)	(1.98–2.26) 2.11 ± 0.12 (6.13)	(1.22–1.51) 1.37 ± 015 (11.72)
longer inflorescence length (mm)	(15.0–30.0) 20.71 ± 3.81 (18.39)	(8.0–16.0) 11.25 ± 2.35 (20.97)	(19–62) 33.66 ± 1.50 (31.20)	(22–25.33) 23.33 ± 1.41 (6.06)	(15.0–34.0) 23 ± 9.84 (42.82)
pedicel length (mm)	(2.97–4.50) 3.58 ± 0.43 (11.95)	(1.23–2.1) 1.60 ± 0.23 (14.83)	(2.0–3.5) 2.73 ± 0.55 (20.12)	(2.23–2.66) 2.40 ± 0.20 (8.37)	(3.7–4.66) 4.05 ± 0.53 (13.10)
bract length of larger bracts (mm)	(6.40–10.50) 7.80 ± 0.97 (12.44)	(3.73–5.83) 4.77 ± 0.45 (9.53)	(5.85–9.73) 7.02 ± 0.86 (12.30)	(5.73–6.76) 6.23 ± 0.45 (7.30)	(6.0–7.33) 6.78 ± 0.69 (10.30)
bracts width of larger bracts (mm)	(3.57–7.40) 4.70 ± 0.78 (16.66)	(2.2–4.66) 2.97 ± 0.49 (16.64)	(3.43–6.93) 4.79 ±0.70 (14.65)	(2.76–3.33) 3.03 ± 0.25 (8.26)	(2.9–6.5) 5.12 ± 1.94 (37.93)
calyx: stipitate glandular hairs	usually absent	absent	usually abundant	abundant	few or absent
calyx tube hairiness (eglandular hairs)	glabrescent (sometimes glabrous)	glabrescent to sparsely hairy	densely hairy	sparsely hairy	sparsely hairy
calyx length (mm)	(6.30–7.66) 6.85 ± 0.33 (4.83)	(3.5–4.442) 3.99 ± 0.20 (5.03)	(4.82–6.36) 5.49 ± 0.40 (7.32)	(5.06–5.44) 5.25 ± 0.21 (4.17)	(6.5–6.74) 6.58 ± 0.13 (2.02)
upper (middle) calyx teeth length (mm)	(1.44–1.98) 1.70 ± 0.19 (10.99)	(0.84–1.26) 1.0 ± 0.10 (10.76)	(1.04–1.54) 1.21 ± 0.13 (10.76)	(1.06–1.12) 1.09 ± 0.02 (2.29)	(2.26–2.36) 2.30 ± 0.05 (2.18)
lower calyx teeth length (mm)	(2.92–3.58) 3.13 ± 0.15 (4.65)	(1.84–2.26) 2.00 ± 0.11 (5.88)	(2.26–2.82) 2.59 ± 0.16 (6.28)	(2.02–2.54) 2.33 ±0.23 (10.23)	(3.24–3.34) 3.29 ± 0.05 (1.52)
length of longer cilia of upper calyx teeth (mm)	(0.02–0.14) 0.08 ± 0.04 (45.74)	(0.02–0.22) 0.12 ± 0.06 (55.23)	(0.52–1.72) 0.68 ± 0.29 (42.65)	(0.04–0.28) 0.21 ± 0.11 (54.11)	(0.2–0.24) 0.22 ± 0.02 (9.09)
length of longer cilia of lower calyx teeth at middle length (mm)	(0.16–0.26) 0.22 ± 0.03 (13.88)	(0.34–0.46) 0.39 ± 0.04 (9.74)	(0.62–0.84) 0.75 ± 0.05 (7.87)	(0.38–0.4) 0.39 ± 0.01 (2.96)	(0.38–0.5) 0.46 ± 0.06 (15.06)
calyx tube length (mm)	(2.34–3.00) 2.61 ± 0.17 (6.48)	(1.42–2.0) 1.61 ± 0.15 (9.88)	(2.04–2.5) 2.32 ± 0.12 (5.28)	(2.1–2.2) 2.16 ± 0.04 (2.04)	(2.5–2.7) 2.59 ± 0.10 (3.88)
length of longer eglandular hair of calyx tube (mm)	(0.16–0.26) 0.22 ± 0.03 (13.11)	(0.28–0.4) 0.34 ± 0.03 (9.95)	(0.6–0.88) 0.74 ± 0.007 (9.90)	(0.3–0.38) 0.33 ± 0.03 (10.19)	(0.24–0.4) 0.30 ± 0.08 (29.05)

Micromorphology was observed on calyces, which were glued directly to aluminium stubs, coated with 40–50 nm gold, and examined with a scanning electron microscopy (SEM) (Zeiss Merlin FE-SEM) at 5 kV.

### ﻿Data analysis

Descriptive statistics (mean, minimum and maximum value, standard deviation and coefficient of variation for each of the studied characters at the taxon level) and univariate statistics (one-way ANOVA followed by Tukey’s test) were calculated to test the significance of differences between taxa within the complex. Overall morphological variation of quantitative traits and relationships of the sampled taxa was evaluated using Principal Component Analysis (PCA). Thymusrichardiisubsp.vigoi was excluded from analysis due to distinct characters in relation to the other taxa (see Identification key). Means of averaged and standardised values of individuals were used as a matrix data in PCA. PCA was computed on the correlation matrix data of all scored traits. The axes with Eigen values > 1 were used in analysis. PCA computation, descriptive and univariate statistics were run in PAST ver. 3.14 (Hammer 2016).

## ﻿Results and discussion

The variation based on SEM micromorphology, univariate and multivariate morphometrics (PCA) of taxa included within *Th.richardii* complex is described and their taxonomic value of the characters is here discussed.

### ﻿Variation of individual quantitative morphological traits within the *Th.richardii* complex

Mean values of the analysed traits go in favour of morphological differentiation among taxa (Table 1). In general, coefficients of variation (CV) did not exceed 20% (Table 1). The values in Th.richardiisubsp.vigoi should be treated with caution because their calculations were based on three individuals. In most cases the coefficients of variation had low (CVs ≤ 10%, 29 cases) and moderate values (CVs from 10 to 20%, 36 cases) (Table 1). High values of coefficients of variation were observed for the trait *length of longer cilia of upper calyx teeth* (LCU) in each taxon and for the trait *longer inflorescence length* (IL). The traits *calyx length* (CL) and *calyx tube length* (CTL) showed the lowest values of coefficients of variation (Table 1).

One-way ANOVA displayed significant differences between mean values of quantitative traits for all subspecies (p ≤ 0.01). The Tukey’s test revealed significant differences among subspecies for the most of the studied traits (Table 2). The highest number of observed differences was 13 (Th.richardiisubsp.ebusitanus vs. Th.humifususvar.aureopunctatus) and 12 (Th.richardiisubsp.richardii vs. Th.humifususvar.aureopunctatus and Th.richardiisubsp.richardii vs. Th.richardiisubsp.nitidus). A considerable level of significant differences (10) was observed between Th.richardiisubsp.richardii vs. Th.richardiisubsp.ebusitanus and Th.richardiisubsp.ebusitanus vs. Th.richardiisubsp.nitidus (9). The smallest number of significant differences (7) was evidenced between Sicilian population of Th.richardiisubsp.nitidus and the Balkan Th.humifususvar.aureopunctatus (Table 2).

**Table 2.** The studied traits differentiating between taxa based on result of the Tukey’s t tests (*p* = 0.01) (abbreviations are as in Table 3).

Taxon	*richardii*	*ebusitanus*	*aureopunctatus*
*ebusitanus*	LL/LW, IL, PL, BL, CL, UTL, LTL, LCU, LCL, CTL, LEH		
*aureopunctatus*	LL, LW, IL, PL, BL, BW, CL, UTL, LTL, LCU, LCL, CTL, LEH	LL, LW, IL, PL, BL, BW, CL, UTL, LTL, LCU, LCL, CTL, LEH	
*nitidus*	LL, LW, LL/LW, PL BL, BW, CL, UTL, LTL, LCL, CTL, LEH	LW, LL/LW, BW, CL, LTL, LCU, LCL, CTL, LEH	LL/LW, IL, PL, BL, CL, LTL, CTL

### ﻿Morphometric relationships among taxa within the *Th.richardii* complex

Morphological variation was explained by three principal components with Eigen values > 1 which clearly separated four morphological clusters corresponding to Th.richardiisubsp.richardii, Th.humifususvar.aureopunctatus, Th.richardiisubsp.ebusitanus and Th.richardiisubsp.nitidus (Table 3, Fig. 1B, C). The first three components accounted for 90.3% of the total variance (PC1 = 57.6%, PC2 = 23.9% and PC3 = 8.8%). The taxa were separated mostly along the first and second axis (Fig. 1B). Thymusrichardiisubsp.nitidus was a neighbouring group to Th.humifususvar.aureopunctatus. A plot onto PC 1 and PC 3 revealed that Th.richardiisubsp.nitidus also represented a distinct cluster within the complex (Fig. 1C). Following characters (CL, LTL, CTL, and BL) with moderate coefficients of correlations were associated with PC1 (Table 3). The characters LEH, LCL and LCU contributed to the PC2. The highest correlations with the PC3 showed derived trait L/W and leaf width (LW) which contributed to the separation of Th.richardiisubsp.nitidus (Table 3). Principal component analysis of quantitative morphological data demonstrates that allopatric populations of the *Th.richardii* complex are clearly distinguishable according to their taxonomic affiliation.

**Table 3.** Principal components revealed by the PCA for the *Thymusrichardii* complex.

Trait		Component		
		PC 1	PC 2	PC 3
LL	leaf (blade) length	0.307	0.032	0.143
LW	leaf (blade) width	0.275	0.181	-0.372
L/W	ratio L/W	0.032	-0.241	0.818
IL	longer inflorescence length	0.246	0.311	0.157
PL	pedicel length	0.321	-0.023	0.088
BL	bract length of larger bracts	0.332	0.036	0.059
BW	bracts width of larger bracts	0.296	0.163	-0.178
CL	calyx length	0.347	-0.062	0.043
UTL	upper (middle) calyx teeth length	0.316	-0.167	-0.094
LTL	lower calyx teeth length	0.339	-0.067	0.033
LCU	length of longer cilia of upper calyx teeth	0.002	0.472	0.250
LCL	length of longer cilia of lower calyx teeth at middle length	-0.108	0.502	0.117
CTL	calyx tube length	0.338	-0.029	0.064
LEH	length of longer eglandular hair of calyx tube	-0.072	0.519	0.098
Eigenvalue		7.990	3.353	1.132
Contribution		0.576	0.239	0.088
Cumulative (%)		0.576	0.815	0.903

Variation in particular morphological traits indicated a similar pattern observed in PCA, confirming a high level of morphological differentiation between the studied taxa. High levels of both morphological and genetic differentiation within plant complexes are not surprising in the Mediterranean. This pattern of variation, which often results in endemism, is particularly pronounced for populations inhabiting the Mediterranean islands (Thompson 2020). Due to different geological and biogeographical processes, long-term isolation, adaptation and specialization to contrasting habitats, the ancestral populations of *Th.richardii* diverged into distinct entities across the Mediterranean and the Balkans.

### ﻿General habit

All the taxa included within the *Thymusrichardii* complex are woody perennials with young or flowering stems with hairs on all faces, more or less evenly distributed. These hairs are eglandular, usually retrorse, up to 0.2 mm long (0.4 mm long in Th.richardiisubsp.ebusitanus), intermixed with sessile glands. According to Morales (2010), the plant length separates the populations from Mallorca from those of Ibiza (7–13 cm vs. 10–24 cm, respectively), but in our opinion this character is rather variable and has no taxonomic significance. We have collected Majorcan plants of Th.richardiisubsp.richardii that measure up to 40 cm in length.

### ﻿Leaves

All the studied taxa have flat leaves, not ciliate at base, with entire margins, except in Th.richardiisubsp.vigoi, which has denticulate leaves. On the basis of leaf morphology (Riera et al. 2007) Th.richardiisubsp.vigoi is easily separable from the rest of the members of the *Th.richardii* complex. Leaves shape varies from ovate to elliptical. Jalas (1972) attributed to Th.richardiisubsp.nitidus leaves more than twice as long as wide. Certainly Th.richardiisubsp.nitidus usually has leaves with a higher length / width ratio than the rest of the taxa (Table 1), but we have studied plants of the island of Marettimo with leaves less than twice as long as wide. On the other hand, some Majorcan specimens of Th.richardiisubsp.richardii have leaves more than twice as long as wide.

The leaves have spheroidal yellowish-reddish glands, and sometimes scattered hairs exist in several taxa of this complex. Some specimens of Th.richardiisubsp.ebusitanus, Th.richardiisubsp.vigoi and Th.richardiisubsp.nitidus have a hairy main midrib in its basal half; this hairiness sometimes extending towards adjacent areas of the blade. Nevertheless, this character seems not to be sufficiently constant for taxonomic purposes.

### ﻿Inflorescence

Flowers are arranged in distinct inflorescences, usually capitate to more or less elongate (up to 62 mm long in Th.richardiisubsp.ebusitanus, Table 1). Bracts are similar to leaves, but smaller, and the bracteoles linear to linear-lanceolate. Pedicels are somewhat longer than documented for the species (Morales 2010), since in Majorcan plants of Th.richardiisubsp.richardii can reach up to 5 mm long.

### ﻿Calyx

Upper calyx-teeth are conspicuously different from lower. The upper lip teeth are usually narrower in Th.richardiisubsp.vigoi. The calyx is green to purplish-green or to purple-violet. This colour variation can be observed within the same population, and the purplish coloration usually occurs in specimens that grow in more exposed places.

**Figure 2.**
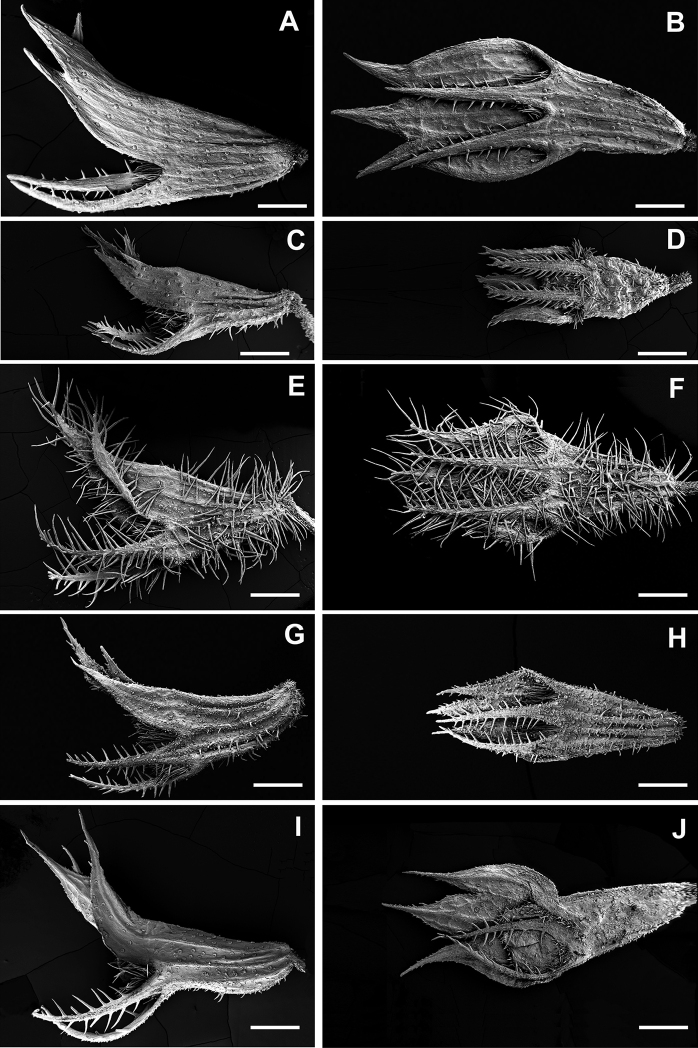
Calyx morphology for *Thymusrichardii* and Th.humifususvar.aureopunctatus. For each taxon lateral (left) and ventral (right) views are shown. Thymusrichardiisubsp.richardii (**A, B** Spain, Mallorca); Th.humifususvar.aureopunctatus (**C, D** Bosnia and Herzegovina, Dužani); Th.richardiisubsp.ebusitanus (**E, F** Eivissa, Ses Balandres); Th.richardiisubsp.nitidus (**G, H** Sicily, Marettimo); Th.richardiisubsp.vigoi (**I, J** Spain, Valencia, La Safor). Scale: 200 micrometres.

Regarding the calyx length, Th.humifususvar.aureopunctatus shows the lowest values, whereas the longest are those of the Majorcan populations of Th.richardiisubsp.richardii (Table 1; Fig. 2). On the other hand, the length of the lower teeth of the calyx also allows separating the previous taxa (Table 1, 2). The presence of shorter calyces in Th.humifususvar.aureopunctatus was documented by Morales (2010), but so far this variation had not been quantified. From our point of view, the calyx length is a diagnostic character to separate the Balkan and the Balearic populations, together with other morphological characters (Table 1, 2).

**Figure 3.**
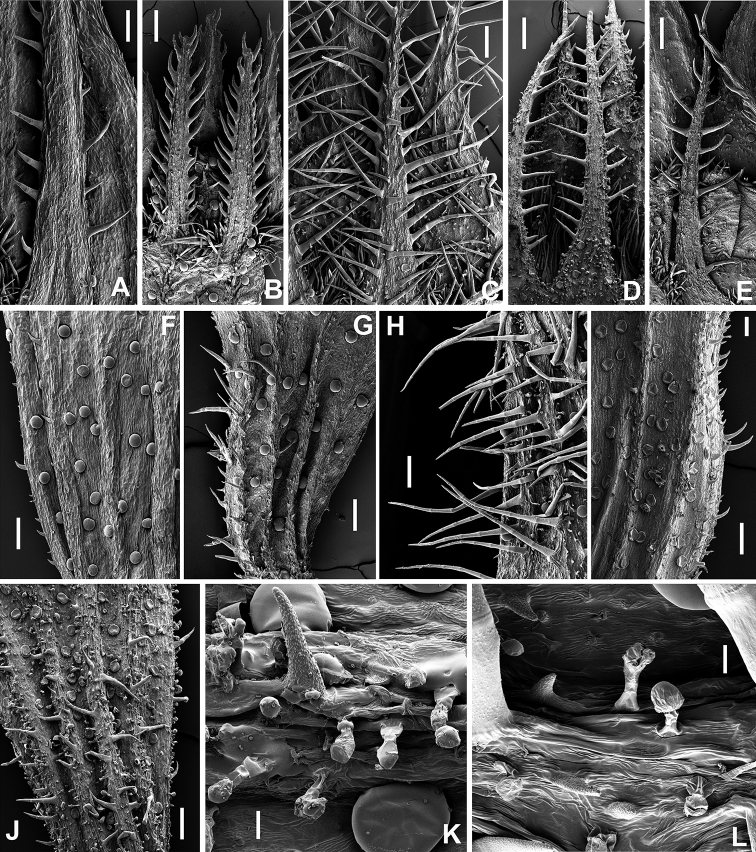
Detail of lower teeth and tube of the calyx and detail of glandular hairs of calyx tube in Thymusrichardiisubsp.richardii (**A, F** Spain, Mallorca, Puig Major, Es Bufador); Th.humifususvar.aureopunctatus (**B, G** Bosnia and Herzegovina, Dužani); Th.richardiisubsp.ebusitanus (**C, H, L** Balearic Island, Eivissa); Th.richardiisubsp.nitidus (**D, J, K** Italy, Sicily, Marettimo); Th.richardiisubsp.vigoi (**E, I** Spain, Valencia, La Safor). Scales: 200 micrometers (**A–J**); 20 micrometers (**K–L**).

The calyces are more or less hairy, with spheroidal yellowish-reddish glands. Our results show that the characters related to the hairiness of the calyx have taxonomic relevance in the *Th.richardii* complex. Calyx indumentum in Th.richardiisubsp.ebusitanus is dense, with long eglandular hairs (up to 1 mm long), mainly on the margins of the lower teeth of the calyx and the ventral part of the calyx tube (Figs 2, 3). On the contrary, the calyx in the Majorcan populations of Th.richardiisubsp.richardii is glabrescent (the upper lip and the dorsal surface of the calyx tube are glabrous or glabrescent) and the hairs are much shorter (Table 1). Thymushumifususvar.aureopunctatus has glabrescent to sparsely hairy calyces, but the eglandular hairs are usually more abundant and longer than in the Majorcan plants of Th.richardiisubsp.richardii.

Stipitate glandular hairs are found in calyces (tube, teeth and even on the adaxial surface of upper teeth) of several taxa (Table 1). As noted by Jalas (1972) these glandular hairs are particularly abundant in Th.richardiisubsp.nitidus (Figs 2, 3). However, stipitate glandular hairs are also usually found (in variable density) in Th.richardiisubsp.ebusitanus, while in Majorca the specimens having these glandular hairs are rather rare but are observed on specimens from Coma de n’Arbona (BC 651145). These glandular hairs were not documented for Balearic plants of *Th.richardii* by Morales (2010). This character seems to be variable in the Balearic populations, since in the same locality there are plants without these glandular hairs.

### ﻿Corolla

The upper lip is emarginate and the lower has 3 subequal lobes (middle lobe somewhat longer). The corolla is more or less hairy on the outer surface, with spheroidal yellowish-reddish glands. Its colour varies from pale rose (sometimes whitish or cream in Th.richardiisubsp.vigoi) to pinkish-purple. The coloration is somewhat variable within the different taxa and in our opinion has no taxonomic significance.

## ﻿Taxonomic treatment

The Majorcan and the Balkan populations, which were included within typical *T.richardii* (Jalas 1971, 1972; Morales 2010) are morphologically distinct; they differ in several characters including calyx size, lower calyx teeth length, length of hairs on the calyx tube, length of pectinate hairs of lower calyx teeth and indumentum density on the calyx (Figs 1–3; Table 1, 3). The Majorcan plants have, compared to those from Bosnia and Herzegovina, longer and less hairy calyces, with shorter hairs and longer lower calyx teeth with shorter (and less dense) pectinate hairs. Examination of herbarium specimens from five populations (16 specimens from Mallorca, 21 from Bosnia and Herzegovina) plus other specimens (see additional specimens examined) revealed that the diagnostic characters are constant within each geographic group. The morphological and biochemical (Llorens et al. 2014) differentiation between the Majorcan and the Balkan populations and their allopatric distribution (they are separated by a gap of ca. 1.300 km) firmly support the recognition of two subspecies, since the level of morphological differentiation between the two taxa does not meet the criteria commonly used to delimit species in *Thymus*. Certainly, further research using molecular markers is needed to reveal genetic relationships and biogeographic history of the *Th.richardii* complex.

### ﻿1. Thymus richardii subsp. richardii

Taxon classificationPlantaeLamialesLamiaceae

Pers., Syn. Pl. 2: 130. 1806 

8DECCD33-118D-5BEB-9A36-0BC03E884E09

≡ Thymusserpyllumvar.richardii (Pers.) Knoche, Fl. Balear. 2: 354. 1922.
≡ Th.serpyllumsubsp.richardii (Pers.) Malag., H. Bianor, Educador Botánico Baleares: 150. 1971.

#### Type.

Holotype (see Rosselló and Sáez 2001: 109): P-Lamarck.

#### Description.

**Figure 4.**
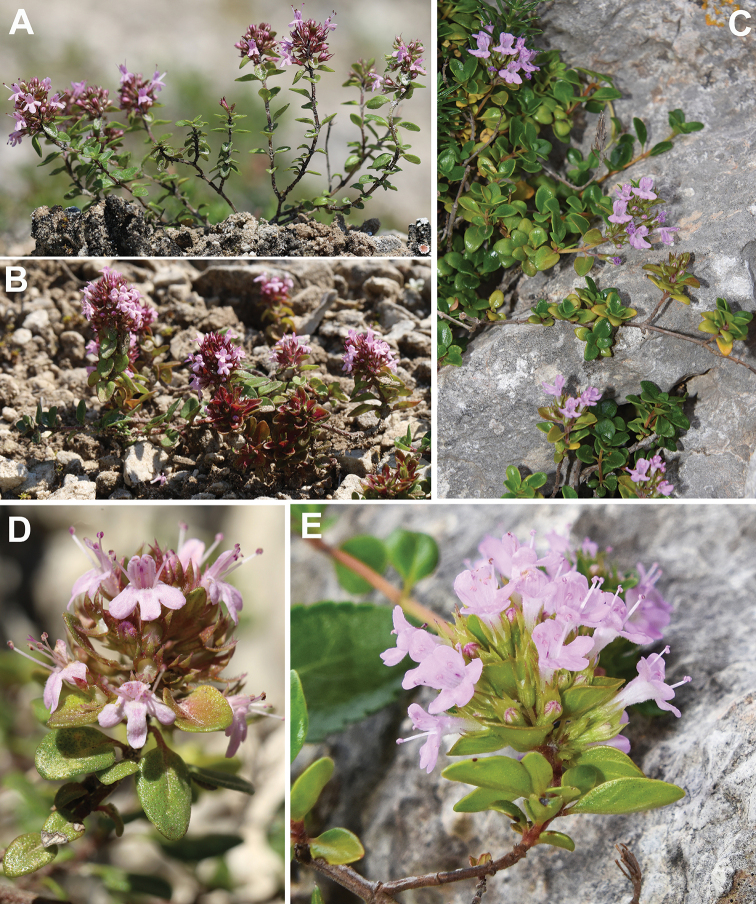
Habit and detail of the inflorescence of Thymushumifususvar.aureopunctatus**A, D** from Bosnia and Herzegovina, Dužani, 3 July 2020; **B** from Bosnia and Herzegovina Džepi, 10 July 2020 (photo F. Bogunić), and of Th.richardiisubsp.richardii**C, E** from Spain, Balearic Islands, Mallorca, Coma de N’Arbona, 17 June 2021 (photo L. Sáez).

Stems up to 47 cm long, procumbent to reptant. Leaf blade up to 13 × 7.7 mm, broadly ovate to elliptical, entire. Inflorescence 15–30 mm long, capitate to oblong; bracts up to 11 × 7.8 mm, similar to leaves, entire, glabrous. Calyx 6–8 mm long, glabrescent (sometimes glabrous), with eglandular hairs up to 0.3 mm long, occasionally with scattered stipitate glandular hairs; calyx tube 2.2–3.2 mm long, glabrescent (sometimes glabrous on the dorsal surface), with eglandular hairs up to 0.3 mm long on the ventral surface; central tooth of upper lip 1.3–2.2 mm long, lower teeth 2.8–3.8 mm long, with pectinate hairs up to 0.3 mm long. Corolla 7–11 mm long, rose to pinkish-purple (Fig. 4, C, E).

#### Chromosome number.

2*n* = 30 (Morales 1995).

#### Distribution.

Endemic to Mallorca, Eastern Balearic Islands (Spain).

#### Habitat.

Cliffs, on humid and north-facing limestone rocks, 250–1430 m a.s.l.

#### Remarks.

This is a rare plant, documented from three localities in the north of Mallorca (Ternelles mountain, Formentor peninsula and Puig Major) of which we have only been able to verify its presence in the last locality, growing on cliffs with very difficult access. This taxon could be facing a population decline. Bianor (1917) at the beginning of the 20^th^ century, considered it as abundant in the Puig Major [“Abondant dans les endroits peu accessibles”]. In fact, there are dozens of specimens from this mountain and which are preserved in various herbaria; mostly collected in the late 19^th^ and early 20^th^ centuries. Currently, Th.richardiisubsp.richardii is very scarce at the same locality where it was reported by Bianor (1917) and the plants are practically inaccessible if climbing techniques are not used. Another population located on a different slope of the same massif is also scarce and very difficult to access. This possible population decline could be due to a loss of potential habitat and intense predation by feral goats (*Caprahircus*).

#### Specimens examined.

**Spain.** Balearic Islands, Mallorca: Comma de n’Arbona, Puig Major, 12 June 1852, *G. Vigineix* (P 04436032, P 04436034); Majorque, 19 June 1869, *Bourgeau* (P 04436046); rochers des Arbonas [n’Arbona], 17 Apr 1870, *F. Barceló* (COI 00045051, P 03389631); Coma de n’Arbona et Puig Major de Son Torrella, 1000–1300 m, 24 June 1885 and 29 July 1885, *Porta & Rigo* (P 04436045); Mallorca: Coma de n’Arbona, Sóller, 30 June 1879, *A. Crespí* (BC 651145, P 04407218); Puig Major, 1000–1400 m, 12 July 1917, *F. Bianor* (BC 50119); Féntes des rochers, Puig Major, 1000–1450 m, 12 July 1918, *F. Bianor* (BC 50118); Puig Major, Féntes des rochers, 1000–1500 m, 7 July 1919, *F. Bianor*, Pl. Espagne F. Sennen 3768 (BC 50123); Coma de n’Arbona, 18 June 1920, *Gros* (BC 859198, P 04407218); Coma de n’Arbona, 4 July, 1936, *Kennedy 48* (BC 103732); Sóller, escletxes dels espadats de la Coma de n’Arbona, July, 1958, *L. Garcías Font* (BC 145169); Puig Major, Coma de n’Arbona, 27 June 1985, *T. Rabassa* (HBJS 5700); Puig Major, Escorca, 10 July 1986, *L. Sáez* (MA 592837); Puig Major de Son Torrella, c. via des Bufador, Escorca, 31SDE8207, 1200 m, 14 June 2006, *L.G. Valle & L. Sáez LS-6445* (L. Sáez, herb. pers.); Escorca, Puig Major, Penyal des Bufador, 31DE8206, 1340 m, 30 June 2020, *L. Sáez* (L. Sáez, herb. pers.); Fornalutx, Coma N’Arbona, 31SDE8105, 1100 m, 17 June 2021, *L. Sáez* (L. Sáez, herb. pers.).

### ﻿2. Thymus richardii subsp. aureopunctatus

Taxon classificationPlantaeLamialesLamiaceae

(Beck) L.Sáez, Bogunić & Bogdanović, comb. & stat. nov.

489497C3-18E2-5994-AEC9-8FDF11379162

urn:lsid:ipni.org:names:77234197-1

≡ Thymushumifususvar.aureopunctatus Beck, Ann. Naturhist. Mus. Wien 2: 142. 1887, basionym.
≡ Th.aureopunctatus (Beck) K. Malý, Prilozi za floru Bosne i Herzegovine: 557. 1908.

#### Type.

**Figure 5.**
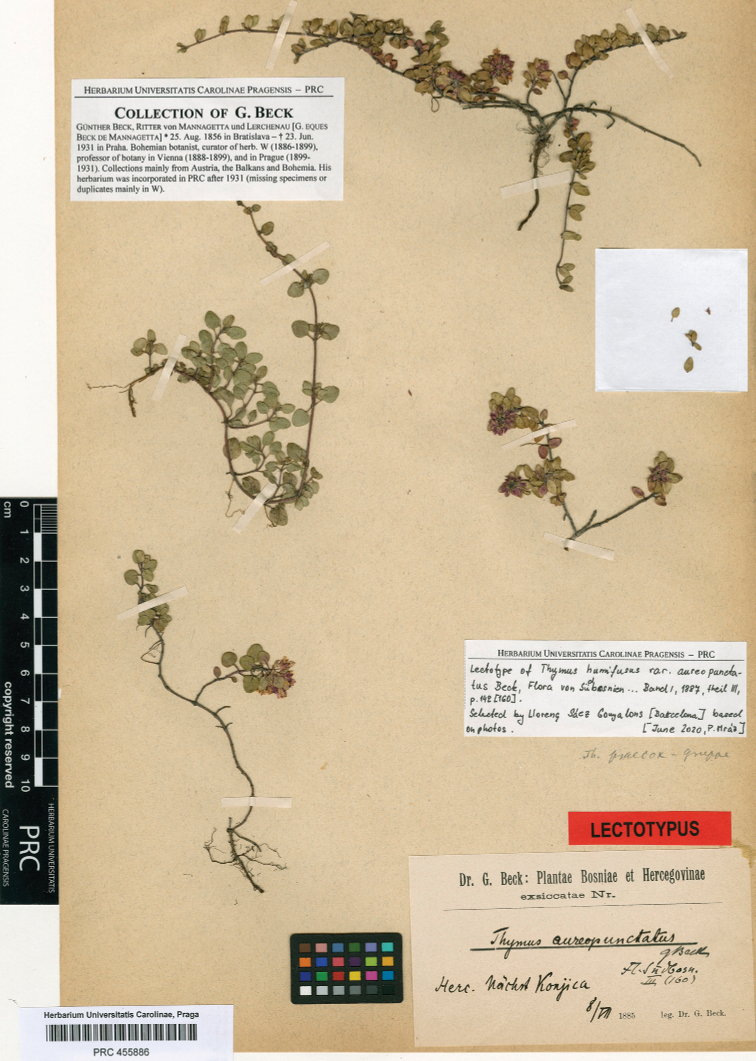
Lectotype of Thymushumifususvar.aureopunctatus (PRC 455886).

Herc. [Herzegovina], Nächst Konjica, 8 July 1885, *G. Beck* (lectotype: PRC 455886! designated here, Fig. 5).

#### Description.

Stems up to 45 cm long, procumbent to reptant. Leaf blade up to 7.7 × 5.3 mm, suborbicular to elliptical, entire. Inflorescence 8–21 mm long, capitate; bracts up to 6.5 × 5 mm, similar to leaves, entire, usually hairy at margin (eglandular hairs up to 1 mm long). Calyx 3–5 mm long, glabrescent to sparsely hairy, with eglandular hairs up to 0.5 mm long, without stipitate glandular hairs; calyx tube 1.3–2.2 mm long, sparsely hairy, with eglandular hairs up to 0.5 mm long on the ventral surface; central tooth of upper lip 0.7–1.6 mm long, lower teeth 2–3 mm long, with hairs pectinate up to 0.5 mm long. Corolla 6–9 mm long, rose (Fig. 4, A, B, D).

#### Chromosome number.

2*n* = 28 (Kaleva 1969).

#### Distribution.

Endemic to surroundings of Konjic (Podorašac, Koznik, Dužani, Dudle, Džepi, Zlatar, Borci, Spiljani, Glavatičevo, Pribilja, Repovica), northern Herzegovina. The taxon covers an area of c. 280 km^2^.

#### Habitat.

Sandy dolomites and dolomitic rocky places, 400–1040 m a.s.l.

#### Remarks.

Beck (1887) described Th.humifususvar.aureopunctatus from “In saxosis prope Konjicam” [Bosnia and Herzegovina] and related this new variety to *Thymushumifusus* Bernh. ex Link, which is currently regarded a synonym of the tetraploid *Th.praecox* Opiz (Jalas 1971; Euro+Med 2006; Plant List 2021; WFO 2021). Günther Beck (1856–1931) was a Bohemian botanist, and his herbarium is currently kept at PRC and W (Stafleu and Cowan 1976). We have been able to locate original material of Th.humifususvar.aureopunctatus at PRC. This is a well-prepared specimen; it matches the description and the provenance indicated in the protologue. Therefore, we designate the specimen with barcode PRC 455886 as the lectotype of the name Th.humifususvar.aureopunctatus (Fig. 5). The taxon occurs in fragmented subpopulations in Bosnia and Herzegovina. Their habitats are threatened by forest succession and canopy closure, but frequent fire incidences represent the most serious threat to its subpopulations. However, the overall population trend of Th.richardiisubsp.aureopunctatus is inferred to be generally stable (F. Bogunić, pers. observ.).

#### Specimens examined.

**Bosnia and Herzegovina**. Konjic, 8 July 1885, *G. Beck* (PRC 455886!, lectoptype); Konjic, Dužani, 43.509894N 18.152114E, 830 m, 10 July 2020, *F. Bogunić* (SARA, ZAGR, L. Sáez herb. pers.); Konjic, Džepi, 43.675506N 18.011992E 757 m, 10 July 2020, *F. Bogunić* (SARA, ZAGR, L. Sáez herb. pers.); Bosnia and Herzegovina, Dudle, 43.540567N 18.121261E, 1034 m, 10 July 2020, *F. Bogunić* (SARA, ZAGR, L. Sáez herb. pers.); Flora Herzegovinae. In pineti (Pinus nigra) inter Pričepa-Bigolje; solo dolomitico, 720 m, 9 August 1908, *K. Maly* (ZA); Flora Hercegovinae. In saxosis dolomiticis ad Repovica prope Konjic, 12 July 1931, *V. Loschingg* (ZA).

### ﻿3. Thymus richardii subsp. ebusitanus

Taxon classificationPlantaeLamialesLamiaceae

(Font Quer) Jalas, Bot. J. Linn. Soc. 64(3): 264. 1971

2A0D0D53-1113-5CC8-A0B3-1705A7C52B0C

≡ Thymusrichardiivar.ebusitanus Font Quer, Cavanillesia 7: 77. 1935;
≡ Th.ebusitanus (Font Quer) Romo, Fl. Silvestres Baleares: 266. 1994.

#### Type.

Eivissa, cala de les Torretes, 29 May 1918, *Font Quer & Gros* (lectotype: BC 50117! designated by Jalas 1971: 264).

#### Description.

Stems up to 54 cm long, more or less reptant to suberect. Leaf blade up to 11 × 8 mm, suborbicular to elliptical, entire. Inflorescence 19–62 mm long, oblong; bracts up to 8.3 × 7.3 mm, similar to leaves, entire; glabrous to hairy at margin and midrib (eglandular hairs up to 1 mm long). Calyx 4.5–6.9 mm long, densely hairy, sometimes hirsute, with eglandular hairs up to 1 mm long, usually with stipitate glandular hairs; calyx tube 1.8–2.7 mm long, densely hairy, with eglandular hairs up to 1 mm long on the ventral surface; central tooth of upper lip 0.9–1.9 mm long, lower teeth 1.9–3.1 mm long, with pectinate hairs up to 1 mm long. Corolla 6–8.5 mm long, pale rose.

#### Chromosome number.

2*n* = 30 (Morales 1990).

#### Distribution.

Endemic to northern Eivissa, Western Balearic Islands (Spain).

#### Habitat.

Limestone rocky places, 5–370 m a.s.l.

#### Specimens examined.

**Spain**. Balearic Islands, Eivissa: cala de les Torretes, 29 May 1918, *Font Quer & Gros* (BC 50117, lectotype); Santa Agnès, a la Cala de les Torretes, 15 June 1918, *Gros* (BC 50116); Cala de’n Damià, 10 July 1920, *Gros* (BC 858975, P 04438273); cala de les Torretes, 8 July 1920, *Gros* (BC 859210, P 04438274); cala de Santa Agnès, 5 m, July 1935, *Gros* (BC 87078, BC87079); Cala Aubarca, 1 Aug 1974, *J.Y. Lesouëf* (MA 620032); vicum Sant Mateu, cala d’Aubarca, 31SCD52, 50 m, 23 June 1979, *Fernández Casas 2883* (BC 633215); Cala den Sardina, 2 June 1981, *Cardona & al*. (BC 644574); Cala den Sardina, 20 June 1983, *L. Llorens* (Herb. Univ. Illes Balears); cingles d’en Recó, 8 June 1997, *N. Torres*, *M. Mayol & L. Sáez* (MA 592780); Ses Balandres, 31SCD5523, 131 m, 3 June 2010, *C. Benedí & L. Sáez* (L. Sáez, herb. pers.).

### ﻿4. Thymus richardii subsp. nitidus

Taxon classificationPlantaeLamialesLamiaceae

(Guss.) Jalas, Bot. J. Linn. Soc. 64: 264. 1971

8108DAB1-BEB2-5D36-B6F7-2521E8F08340

≡ Thymusnitidus Guss., Fl. Sicul. Syn. 2(1): 97. 1844;
≡ Th.serpyllumvar.nitidus (Guss.) Bég. in Fiori & Béguinot, Fl. Italia 3: 66. 1903. - Th.sensu lucidus Guss., Fl. Sicul. Prodr., Suppl.: 198. 1843

#### Type.

Marettimo, 10 May 1829, Herb. Gussone Sicilia *s.c*., bottom-right specimen (Lectotype: NAP-Gussone!, designated by Bartolucci et al. 2013: 1310).

#### Description.

Stems up to 25 cm long, procumbent or suberect. Leaf blade up to 10 × 4.5 mm, elliptical, entire. Inflorescence 8–30 mm long, subcapitate to oblong; bracts up to 7 × 4 mm, similar to leaves, entire, glabrous to hairy at margin and midrib (eglandular hairs up to 0.4 mm long, mixed with stipitate glandular hairs). Calyx 4.5–6.3 mm long, densely covered by stipitate glandular hairs and sparse eglandular hairs up to 0.5 mm long; calyx tube 1.9–2.5 mm long, with eglandular hairs up to 0.5 mm long on the ventral surface; central tooth of upper lip 0.8–1.5 mm long, lower teeth 2–3 mm long, with pectinate hairs up to 0.5 mm long. Corolla 6.5–9.5 mm long, pale rose.

#### Chromosome number.

2*n* = 28 (Morales 1997)

#### Distribution.

Endemic to Island of Marettimo, Sicily (Italy).

#### Habitat.

Limestone rocky places, 10–600 m a.s.l.

#### Specimens examined.

**Italy.** Sicily, Marettimo, *sine leg*. (PAL); Isola di Marettimo, rupi di P. Anzine, 21 July 2007, *Scuderi* (VAL 184304).

### ﻿5. Thymus richardii subsp. vigoi

Taxon classificationPlantaeLamialesLamiaceae

Riera, Güemes & Rosselló, Fl. Montiber. 37: 78. 2007

0D988EF0-69CF-5B18-96B2-A0B427C1A8C1

Type. Spain, Valencia, Villalonga, La Safor, ad l’Orxa, 30SYJ3706, 600 m, 4 July 2000, *J. Riera & J. Güemes* (holotype: VAL 185406!; isotype: MA 757804!; Riera et al. 2007).
= Thymusrichardiivar.valentinus O. Bolòs & Vigo, Collect. Bot. (Barcelona) 14: 95. 1983

#### Type.

Spain, Valencia province, Valentia, c. Gandia, 15 Sept 1950, *P. Cañigueral* (holotype: BC 119858!; Bolòs and Vigo 1983).

#### Description.

Stems up to 16 cm long, suberect to erect. Leaf blade up to 11 × 8.1 mm, ovate-triangular, denticulate. Inflorescence 15–34 mm long, usually oblong; bracts up to 8 × 7 mm, similar to leaves, denticulate, usually glabrous. Calyx 5.8–7 mm long, sparsely hairy, with eglandular hairs up to 0.7 mm long, sometimes with sparse stipitate glandular hairs; calyx tube 2.1–2.9 mm long, with eglandular hairs up to 0.5 mm long on the ventral surface; central tooth of upper lip 1.8–2.6 mm long, lower teeth 3–3.5 mm long, with pectinate hairs up to 0.7 mm long. Corolla 7–10 mm long, whitish to pale rose, sometimes cream.

#### Chromosome number.

Unknown.

#### Distribution.

Endemic to Alicante and Valencia provinces (Spain).

#### Habitat.

Open scrub, on limestone soil, 130–600 m a.s.l.

#### Remarks.

Plants which were considered to be hybrids between Th.richardiisubsp.vigoi and *T.piperella* L. have been called *T.×bolosii*. The hybrid has been reported from a small area of Serra de la Safor, eastern Spain (Riera et al. 2020).

#### Specimens examined.

**Spain.** Alicante province: La Vilallonga, La Safor, 136 m, 22 June 1984, *J.B. Peris & G. Stübing* (BC 674556); Valencia province, Valentia, c. Gandia, 15 Sept 1950, *P. Cañigueral* (BC 119858); Villalonga, La Safor, ad l’Orxa, 30SYJ3706, 600 m, 4 July 2000, *J. Riera & J. Güemes* (VAL 185406!).

### ﻿Identification key for *Thymusrichardii* complex

We propose the following key for the subspecies of the *Thymusrichardii* complex in order to include the new proposed subspecies.

**Table:** 

1	Leaves denticulate, cordate at base	**subsp.vigoi**
–	Leaves entire (rarely slightly denticulate) cuneate at base	**2**
2	Calyx glabrescent (sometimes glabrous) to sparsely hairy; glandular hairs usually scarce or absent	**3**
–	Calyx hairy to densely hairy; glandular hairs usually present	**4**
3	Calyx 3–5 mm long	**subsp.aureopunctatus**
–	Calyx 6–8 mm long	**subsp.richardii**
4	Calyx with abundant eglandular hairs, usually with sparse to dense glandular stipitate hairs; lower calyx teeth with pectinate pluricellular eglandular hairs up to 1 mm long	**subsp.ebusitanus**
–	Calyx densely covered by glandular hairs, mixed with sparse eglandular hairs; calyx teeth with pectinate eglandular pluricellular hairs up to 0.5 mm long	**subsp.nitidus**

## Supplementary Material

XML Treatment for Thymus richardii subsp. richardii

XML Treatment for Thymus richardii subsp. aureopunctatus

XML Treatment for Thymus richardii subsp. ebusitanus

XML Treatment for Thymus richardii subsp. nitidus

XML Treatment for Thymus richardii subsp. vigoi

## ﻿Appendix I. List of specimens included in morphometric analyses.

***Thymusrichardii*** subsp. ***richardii*. Spain.** Mallorca: Coma de n’Arbona, Sóller, 30 June 1879, *A. Crespí* (BC 651145) [1 specimen]; Fentes des rochers, 1000–1150 m, 12 July 1918, *Bianor* (BC 50118) [1 specimen]; *Ibidem* 7 July, 1919, *Bianor* (BC 50123) [1 specimen]; *Ibidem* 12 July, 1917, *Bianor* (BC 50119) [1 specimen]; Coma de n’Arbona, 18 June 1920, *Gros* (BC 859198) [1 specimen]; Col de n’Arbona, 4 July, 1936, *Kennedy 48* (BC 103732) [1 specimen]; Espadats de la coma de n’Arbona, July, 1958, *Garcias Font* (BC 145169) [1 specimen]; Coma de n’Arbona, 27 June 1985, *T. Rabassa* (HBJS 5700) [1 specimen]; Coma N’Arbona, 31SDE8105, 1100 m, 17 June 2021, *L. Sáez* (L. Sáez, herb. pers.) [4 specimens]. Puig Major, via des Bufador, Escorca, 31SDE8207, 1200 m, 14 June 2006, *L.G. Valle & L. Sáez LS-6445* (L. Sáez, herb. pers.) [1 specimen]; Puig Major, Penyal des Bufador, 31DE8206, 1330–1350 m, 30 June 2020, *L. Sáez* (L. Sáez, herb. pers.) [8 specimens].

***Thymusrichardii*** subsp. ***ebusitanus.* Spain.** Balearic Islands, Eivissa: cala de les Torretes, 29 May 1918, *Font Quer & Gros* (BC 50117, lectotype) [2 specimens]; Santa Agnès, a la Cala de les Torretes, 15 June 1918, *Gros* (BC 50116) [1 specimen]; cala de les Torretes, 8 July 1920, *Gros* (BC 859210) [1 specimen]; Cala de’n Damià, 10 July 1920, *Gros* (BC 858975) [1 specimen]; cala de Santa Agnès, 5 m, July 1935, *Gros* (BC 87078, 87079) [2 specimens]; vicum Sant Mateu, cala d’Aubarca, 31SCD52, 50 m, 23 June 1979, *Fernández Casas 2883* (BC 633215) [1 specimen]; Cala den Sardina, 2 June 1981, *Cardona & al*. (BC 644574) [1 specimen]; Cala den Sardina, 20 June 1983, *L. Llorens* (Herb. Univ. Illes Balears) [1 specimen]; Ses Balandres, 31SCD5523, 131 m, 3 June 2010, *C. Benedí & L. Sáez* (L. Sáez, herb. pers.) [5 specimens].

***Thymusrichardii*** subsp. ***aureopunctatus*. Bosnia and Herzegovina.** Konjic, Dužani, 43.509894N 18.152114E, 830 m, 10 July 2020, *F. Bogunić* (SARA, ZAGR, L. Sáez herb. pers.) [7 specimens]; Konjic, Džepi, 43.675506N 18.011992E 757 m, 10 July 2020, *F. Bogunić* (SARA, ZAGR, L. Sáez herb. pers.); [7 specimens]; Dudle, 43.540567N 18.121261E, 1034 m, 10 July 2020, *F. Bogunić* (SARA, ZAGR, L. Sáez herb. pers.) [7 specimens].

***Thymusrichardii*** subsp. ***nitidus*. ITALY.** Sicily, Marettimo s.d., s.r. (PAL) [3 specimens]; Isola di Marettimo, rupi di P. Anzine, 21 July 2007, *Scuderi* (VAL 184304) [1 specimen].
